# Impact of forage-free diet with cottonseed cake and of feeding frequency using an automated system on productivity and profitability in feedlot-finished cattle

**DOI:** 10.1007/s11250-026-04904-2

**Published:** 2026-02-24

**Authors:** Maria Fernanda Garcia Baveloni, Luís Carlos Vinhas Ítavo, Luan Sousa dos Santos, Priscilla Dutra Teixeira, Camila Celeste Brandão Ferreira Ítavo, Marina de Nadai Bonin Gomes, Adrianni Dias Borges, João Victor Souza de Oliveira, Lívia do Nascimento Gomes, Guilherme Zanoni Baratella, Rodolpho Martin do Prado

**Affiliations:** 1 Faculty of Veterinary Medicine and Animal Science of the Federal University of Mato Grosso do Sul, Av. Senador Filinto Muller, 2443, Vila Ipiranga, Campo Grande, MS CEP 79070-900 Brazil; 2 Faculty of Agricultural and Food Sciences of Laval University, Paul-Comtois Pavilion 2425, Rue de l’Agriculture, Local 1122, Quebec, G1V 0A6 Canada

**Keywords:** Automated system, Forage-free diet, Feeding frequency, Cottonseed cake

## Abstract

We hypothesized that a forage-free diet, with cottonseed cake as the fiber source and administered six times daily using an automated system by a feeder robot, could optimize the productive and economic performance of feedlot-finished cattle. Thus, the objective of this study was to evaluate the effects of fiber source and feeding frequency on the productive and economic performance of beef cattle finished in a feedlot. Twenty-four uncastrated, 24-month-old Nellore young bulls, averaging 462 ± 23.44 kg in initial live weight were used in a completely randomized design with a 2 × 2 factorial scheme incorporating two fiber sources (corn silage or cottonseed cake) and two feeding frequencies (twice or six times per day) with six replicates of each. Replacing silage with cottonseed cake did not affect productive performance (*P* > 0.05). However, it resulted in lower feed costs (*P* = 0.003) and a higher net margin per animal (*P* = 0.02). Twice-daily automated feeding increased nutrient intake (*P* < 0.05) and showed a trend toward higher average daily gain (*P* = 0.06). Rumination time was longer with a forage-based diet (*P* < 0.05), whereas rumination efficiency was higher with a forage-free diet (*P* = 0.02). Cottonseed cake is a viable alternative to forage-free diets in feedlots. However, increasing feeding frequency did not provide additional benefits.

## Introduction

Due to their higher energy content, the use of high-concentrate or even forage-free diets has been widely adopted to optimize animal performance, reduce operational costs, and increase feed efficiency in feedlot-finished cattle (Millen et al. 2009; Pinto and Millen 2018; Silvestre and Millen 2021). However, these diets can lead to increased rumen fermentation, which can result in metabolic disorders and dysfunction, and consequently, reduce dry matter intake (DMI) and animal performance (Owens et al. 1998; Mertens 1997; Nagaraja and Lechtenberg 2007).

In this sense, incorporating fibrous by-products such as cottonseed cake into the diet can encourage rumination and stabilize rumen pH. These by-products provide adequate levels of physically effective neutral detergent fiber (peNDF) and are low-cost (Goulart et al. 2020a; Arcanjo et al. 2022; Valadares Filho et al. 2016). To minimize the negative impacts of a concentrate-rich diet, management strategies such as increasing the frequency of feed supply are advantageous.

Feeding frequency directly affects rumen fermentation dynamics, volatile fatty acid production, and rumen papillae morphology (Sutton et al. 1986; Jonker et al. 2014; Silva et al. 2018). Frequent and regular feeding minimizes fluctuations in DMI and sudden drops in rumen pH, creating a more favorable environment for rumen microbiota (Schwartzkopf-Genswein et al. 2003; Saldanha et al. 2021). However, higher feeding frequencies increase operational costs due to the greater demand for labor (Da Borso et al. 2017; Romano et al. 2023). One alternative is to use a feeder robot to automate the feeding process in feedlots.

To mitigate this challenge, automated feeding systems have been adopted for feedlot cattle. Similar to systems already in place for pig farming (Romano et al. 2023), these systems provide precise and regular feeding, reduce labor dependency, and optimize nutritional management. Automating feed through management reduces variability associated with human evaluation and optimizes feed allocation, thereby improving feed efficiency and animal performance. Furthermore, implementing this automated system decreases dependence on specialized labor and enables more precise and consistent nutritional management in feedlots (McMeniman et al. 2025).

Based on the above information, we formulated the hypothesis that a forage-free diet, with cottonseed cake as the fiber source and administered six times daily using an automated system by a feeder robot, could optimize the productive and economic performance of feedlot-finished cattle. Thus, this study aimed to evaluate the effects of automated feeding frequency and an alternative fiber source on the intake, productivity, economics, feed efficiency, and carcass characteristics of feedlot-finished beef cattle.

## Materials and methods

The experiment was carried out in the beef cattle feedlot sector at the School Farm of the Faculty of Veterinary Medicine and Animal Science of the Federal University of Mato Grosso do Sul (FAMEZ/UFMS), located in the municipality of Terenos, Mato Grosso do Sul, Brazil, at longitude 20 ° 26’ 43.9” S and longitude 54°50’44.2” W. The region’s climate is tropical, predominantly humid to sub-humid. The rainfall varies from 1,500 to 1,750 mm annually and is regular, with a dry period, of less than four months, corresponding to a water deficiency of 350–500 mm., and the daily maximum temperatures can exceed 35 °C. The experiment was conducted from October to December 2024.

The experimental procedures were submitted for review and approved by the Animal Use Ethics Committee of the Federal University of Mato Grosso do Sul (Protocol No. 1,181/2021).

### Animals, experimental design, and diets

Twenty-four uncastrated Nellore young bulls from the beef cattle sector of the FAMEZ/UFMS School Farm, with an average initial live weight of 462 ± 23.44 kg, were housed in individual pens in a completely randomized design with four treatments (six animals per treatment). The animals were housed in individual stalls (4 × 20 m) with concrete trough floors, individual feeders (2.5 m), and a waterer divided between two stalls.

The experimental period lasted 76 days and included a 15-day adaptation phase for feeding management and diets. During this period, the experimental diet was used, with feed restricted by 50% on the first day and then increased by 5% each subsequent day. At the beginning of the adaptation, the animals were treated for internal and external parasites with a dewormer (Cydectin; Zoetis Veterinary Medicine Industry Ltda., Campinas, SP, Brazil) and vaccinated against clostridial diseases (Excell 10; Dechra Brasil Veterinary Products Ltda., Londrina, PR, Brazil), and respiratory diseases (Inforce 3; Zoetis Veterinary Medicine Industry Ltda., Campinas, SP, Brazil).

The experimental diets (Table 1) were formulated to be isonitrogenous and isoenergetic, following BR-Corte guidelines (Valadares Filho et al. 2016), to meet the nutritional requirements for an expected average daily gain (ADG) of 1.6 kg/day. The diets were differentiated according to fiber source: one contained corn silage as roughage, and the other used cottonseed cake as an alternative source of peNDF (Table 2). To achieve the target crude protein concentration in the diets, urea was incorporated into the corn silage treatment to compensate for its lower protein content relative to cottonseed cake. Urea was thoroughly mixed with the silage before it was delivered to the feed bunk.

**Table 1:** Diets for finishing Nellore young bulls in confinement according to fiber source

	Treatment	
	Diet with roughage	Forage-free diet
Whole plant corn silage	33.2	-
Cottonseed cake		36.0
Concentrate^#^	64.2	64.0
Extruded urea	2.60	-
Chemical composition (%)		
Dry matter (% of fresh weight)	70.7	92.4
Organic matter	92.7	93.5
Ashes	7.30	6.40
Crude protein	18.7	18.8
Neutral detergent fiber	37.5	37.5
Ether extract	2.60	4.80
Total digestible nutrients^1^	69.6	69.6
Cost (U$/fresh diet)	2.51	3.08

Fiber source: Whole plant corn silage (282 g/kg DM; 965 g/kg OM; 67.6 g/kg CP; 330 g/kg NDF) and cottonseed cake (889 g/kg DM; 955 g/kg OM; 255 g/kg CP; 454 g/kg NDF)

^1^Estimated by equation TDN = 91.0246–0.571588 × NDF (Cappelle et al. 2001)

^#^Concentrate: 70.75% of ground corn, 22% of soybean meal; 1.25% of urea, 4% of calcium carbonate, and 2% of mineral premix. Guarantee levels: 880 g/kg DM; 180 g/kg CP; 56 g/kg NPN; 720 g/kg TDN; 120 g/kg NDF; 238 g/kg ADF; 95 g/kg ash; 25 g/kg EE; 12 g/kg Ca; 3.5 g/kg P; 3.0 g/kg S; 3.5 g/kg Na; 2.0 g/kg Mg; 100 mg/kg Zn; 30 mg/kg Cu; 1 mg/kg Co; 1 mg/kg I; 40 mg/kg Mn; 0.7 mg/kg Se

**Table 2:** Fresh matter, dry matter, and neutral detergent fiber retained in the compartments of the *Penn state particle separator* sieve assembly from the corn silage and cottonseed cake fiber sources

	Whole plant corn silage				Cottonseed cake			
	19 mm	8 mm	4 mm	< 4 mm	19 mm	8 mm	4 mm	< 4 mm
Fresh matter (g/kg)	32.0	580	143	48.0	40.0	548	124	64.0
Fresh matter (%)	4.00	72.2	17.8	6.00	5.10	70.6	16.0	8.30
Dry matter (g/kg)	8.00	159	42.0	14.00	36.0	493	111	57.0
DM content of retained material (g/kg)	273	300	323	316	963	951	957	959
Neutral detergent fiber (g/kg)	11.0	191	47.0	16.0	18.0	247	56.0	29.0
NDF content of retained material (g/kg)	492	397	332	281	524	576	546	483

Starting material: 803 g of fresh matter from whole plant corn silage (peNDF = 762 g/kg of NDF), and 776 g fresh matter from cottonseed cake (peNDF = 757 g/kg of NDF)

The design used was completely randomized in a 2 × 2 factorial scheme, with two fiber sources (corn silage or cottonseed cake) and two feeding frequencies (twice or six times a day), totaling four treatments with six replicates per treatment. The treatments consisted of the following: (1) a diet of corn silage and concentrate, supplied twice a day; (2) a diet of corn silage and concentrate, supplied six times a day; (3) a forage-free diet containing cottonseed cake, supplied twice a day; (4) a forage-free diet containing cottonseed cake, supplied six times a day. These diets were provided either twice daily (at 08:30 and 15:30) or six times daily (at 08:30, 09:30, 10:30, 13:30, 14:30, and 15:30), after any leftovers had been collected. In the corn silage-based diet, roughage was manually provided once daily, while the concentrate was delivered using an automated feeder. In contrast, for the forage-free diet containing cottonseed cake, the total mixed ration was entirely supplied by the automated feeding system.

### Nutrient intake and performance

Nutrient intake was determined by the difference between the amount fed and the amount present in the leftovers, which were considered waste. Based on this value, daily adjustments were made to the diet supply, increasing or reducing the total feed amount by 5% daily, depending on the level of leftovers observed in the pens.

The animals were weighed on days 0, 15, 30, 60, and 76 of confinement, following a 16-hour fasting period and before feeding, to monitor animal gain. Total weight gain (TWG) was determined by the difference between final body weight (BW) and initial BW. ADG was calculated by dividing TWG by the number of days on feed. Feed efficiency (FE) was expressed as the ratio of ADG to average daily DMI. Feed conversion (FC) was calculated by the ratio of average DMI to ADG.

To determine the peNDF content of the fiber sources, corn silage and cottonseed cake, the Penn State Particle Separator sieve set was used. The peNDF was calculated based on the proportion of neutral detergent fiber (NDF) retained on the 19 mm and 8 mm sieves.

### Blood analysis

Blood samples were collected by coccygeal vein puncture at the beginning and end of the experimental period, using tubes containing EDTA as an anticoagulant. The samples were then centrifuged at 2,000 × g for 10 min at room temperature. The plasma obtained was frozen at − 20 °C for later analysis of the following biochemical parameters: glucose (kit ref. 04657527), aspartate aminotransferase (AST; kit ref. 10745120), gamma-glutamyltransferase (GGT; kit ref. 05401461), urea (kit ref. 11200666), and total protein (kit ref. 04657586). The analyses were performed at the Clinical Analysis Laboratory of the FAMEZ/UFMS Veterinary Hospital in Campo Grande, MS, Brazil.

### Ingestive behavior

The assessments of the animals’ ingestive behavior were conducted monthly over a continuous 24-hour period, analyzing both the diet supply period (from 8:30 to 15:30) and behavior throughout the day. Observations were made visually by a team of observers, with each observer monitoring six pens and recording feeding, water intake, rumination, and idle behaviors at five-minute intervals. The analyzed variables included the time spent on feeding, water intake, rumination, and idleness (minutes/day), with averages calculated for both periods. Feeding efficiency was assessed by dividing DMI by feeding time, and rumination efficiency was calculated by dividing NDF intake by rumination time.

### Slaughter and carcass evaluation

At the end of the experimental period, the animals were fasted for 16 h and slaughtered at a commercial slaughterhouse using the cerebral concussion technique, followed by division of the jugular vein, removal of the hide, and evisceration. Hot carcass weights (HCW) were determined immediately after evisceration. Carcass yield (CY) was calculated as proposed by Gomes et al. (2021).

### Economic analysis

The average feed cost for the steers was calculated based on the total amount of ration used (including the total diet, fiber source, and concentrate) over the 76-day period, the animals’ BW, and their weight gain. The cost per ton of raw material was US$308.06 for the roughage-free diet and US$251.61/ton for the corn silage diet. The economic analysis incorporated the carcass-equivalent value at slaughter (US$5.61 per kg). The revenue per animal (US$) was calculated by multiplying the carcass weight by the carcass price. Finally, the net margin per animal was determined as the difference between the gross revenue from the animal’s sale and the total feed cost incurred during the feedlot period.

### Laboratory analysis

The analyses of food samples, complete diet, and leftovers were performed at the Applied Nutrition Laboratory of the FAMEZ/UFMS. To determine the chemical composition, the samples were pre-dried in a forced ventilation oven at 55 °C for 72 h and ground in a mill with a 1-mm sieve. The samples were analyzed as described by Detmann et al. (2021): dry matter (DM; INCT method G-003/1); ash (INCT method M-001/2), organic matter (OM; INCT method M-001/2); ether extract (EE; INCT method G-004/1), neutral detergent insoluble fiber (NDF; INCT method F-001/2), crude protein (CP; total *N* × 6.25; INCT method N-001/2). The estimated total digestible nutrients (TDN) were calculated according to Cappelle et al. (2001), using the equation: TDN = 91.0246–0.571588×NDF.

### Statistical analysis

The data were analyzed using the GLM (General Linear Model) procedure of the SAS Studio 3.8 software, executed on the SAS 9.4 platform (SAS Institute Inc., Cary, NC, USA), with the treatments considered as a fixed effect. The statistical model used was as follows:

$$\:Yijk\hspace{0.17em}=\hspace{0.17em}\mu\:\hspace{0.17em}+\hspace{0.17em}FSi\:+\:FFj\:+\:FS\times\:FFk\:+\:eijk$$

Where Y_ijk_ is the observed value of animal k of fiber source i at feeding frequency j; µ is the overall mean; FS_i_ is the effect of fiber source (i = 1, 2; being corn silage or cottonseed cake); FF_j_ is the effect of feeding frequency j (j = 1, 2; being supplied twice or six times per day); FS×FF_k_ is the effect of the interaction between fiber source and feeding frequency, and e_ijk_ is the random error associated with each observation.

The P-values were considered significant when *P* ≤ 0.05, and trends were considered when the interval was 0.05 < *P* ≤ 0.10.

## Results

No significant interaction was observed between fiber source and feeding frequency about dry matter and nutrient intake (*P* ≥ 0.50; Table 3). However, an effect of diurnal feeding frequency on these variables was observed. The animals fed twice a day showed higher DMI (+ 2.5 kg/day; *P* = 0.006), OM intake (*P* = 0.006), CP intake (*P* = 0.006), EE intake (*P* = 0.02), NDF intake (*P* = 0.006), peNDF intake (*P* = 0.006), and TDN intake (*P* = 0.006) than animals fed six times a day. Fiber source did not significantly influence DMI (kg/day or %BW; *P* > 0.12), but animals fed forage-free diet had higher EE intake (*P* = 0.0001). There were no significant differences for the other nutrients (*P* ≥ 0.11).

**Table 3:** Intake of Nellore young bulls finished in confinement according to the dietary fiber source and feeding frequency

	Treatment				SEM	*P*-value		
	Diet with roughage		Forage-free diet			FS	FF	FS×FF
	Twice times per day (2x)	Six times per day (6x)	Twice times per day (2x)	Six times per day (6x)				
DMI (kg/day)	11.7^a^	8.79^b^	10.3^a^	8.22^b^	0.82	0.22	0.006	0.59
DMI (%BW)	2.21^a^	1.73^b^	1.96^a^	1.62^b^	0.11	0.12	0.001	0.54
OMI (kg/day)	11.0^a^	8.25^b^	9.58^a^	7.68^b^	0.77	0.20	0.006	0.58
CPI (kg/day)	2.17^a^	1.63^b^	1.93^a^	1.55^b^	0.15	0.27	0.006	0.60
EEI (kg/day)	0.31^Ba^	0.24^Bb^	0.53^Aa^	0.42^Ab^	0.03	0.0001	0.02	0.77
NDFI (kg/day)	4.38^a^	3.30^b^	3.85^a^	3.08^b^	0.31	0.22	0.006	0.59
NDFI (%BW)	0.83^a^	0.65^b^	0.74^a^	0.61^b^	0.04	0.11	0.001	0.50
peNDFI (kg/day)	3.32^a^	2.54^b^	2.91^a^	2.32^b^	0.23	0.19	0.006	0.59
TDNI (kg/day)	8.14^a^	6.12^b^	7.14^a^	5.72^b^	0.57	0.22	0.006	0.59

FS: fiber source, FF: feeding frequency; FS × FA: fiber source × feeding frequency interaction; SEM = standard error of the mean

DMI = dry matter intake; OMI = organic matter intake; CPI = crude protein intake; EEI = ether extract intake; NDFI = neutral detergent fiber intake; peNDFI = physically effective neutral detergent fiber intake; TDNI = total digestible nutrients intake

^#^Means followed by a lowercase letter on the same line differ from each other by the F test for feeding frequency and means followed by an uppercase letter on the same line differ from each other by the F test for fiber source

There was no significant interaction between fiber source and daytime feeding frequency, and there were no significant effects of fiber source on the productive performance variables (*P* ≥ 0.20; Table 4). However, animals fed twice a day tended to have greater TWG (+ 29.0 kg; *P* = 0.06) and higher ADG (+ 0.4 kg/day; *P* = 0.06) than those fed six times a day. Carcass characteristics, such as HCW and CY, were unaffected by the treatments (*P* > 0.10).

**Table 4:** Productive performance of Nellore young bulls finished in confinement according to the dietary fiber source and feeding frequency

	Treatment				SEM	*P*-value		
	Diet with roughage		Forage-free diet			FS	FF	FS×FF
	Twice times per day (2x)	Six times per day (6x)	Twice times per day (2x)	Six times per day (6x)				
Initial BW (kg)	465	462	462	459	16.8	0.85	0.86	0.99
Final BW (kg)	591	561	576	542	28.1	0.53	0.25	0.95
TWG (kg)	127^a^	99.4^b^	114^a^	83.6^b^	15.0	0.33	0.06	0.92
ADG (kg/day)	1.67^a^	1.31^b^	1.50^a^	1.10^b^	0.20	0.33	0.06	0.92
HCW (kg)	322	294	299	305	17.0	0.70	0.51	0.31
CY (%)	54.2	53.0	52.0	56.2	0.02	0.81	0.47	0.20
FC	7.00	6.71	6.87	7.47	0.71	0.44	0.47	0.46
FE	0.14	0.15	0.14	0.13	0.01	0.48	0.66	0.51

FS: fiber source, FF: feeding frequency; FS × FA: fiber source × feeding frequency interaction; SEM = standard error of the mean

BW = body weight; TWG = total weight gain; ADG = average daily gain; HCW = hot carcass weight; CY = carcass yield; FC = feed conversion (DMI/ADG); FE = Feed efficiency (ADG/DMI)

^#^Means followed by a lowercase letter on the same line differ from each other by the F test

**Fig. 1:**
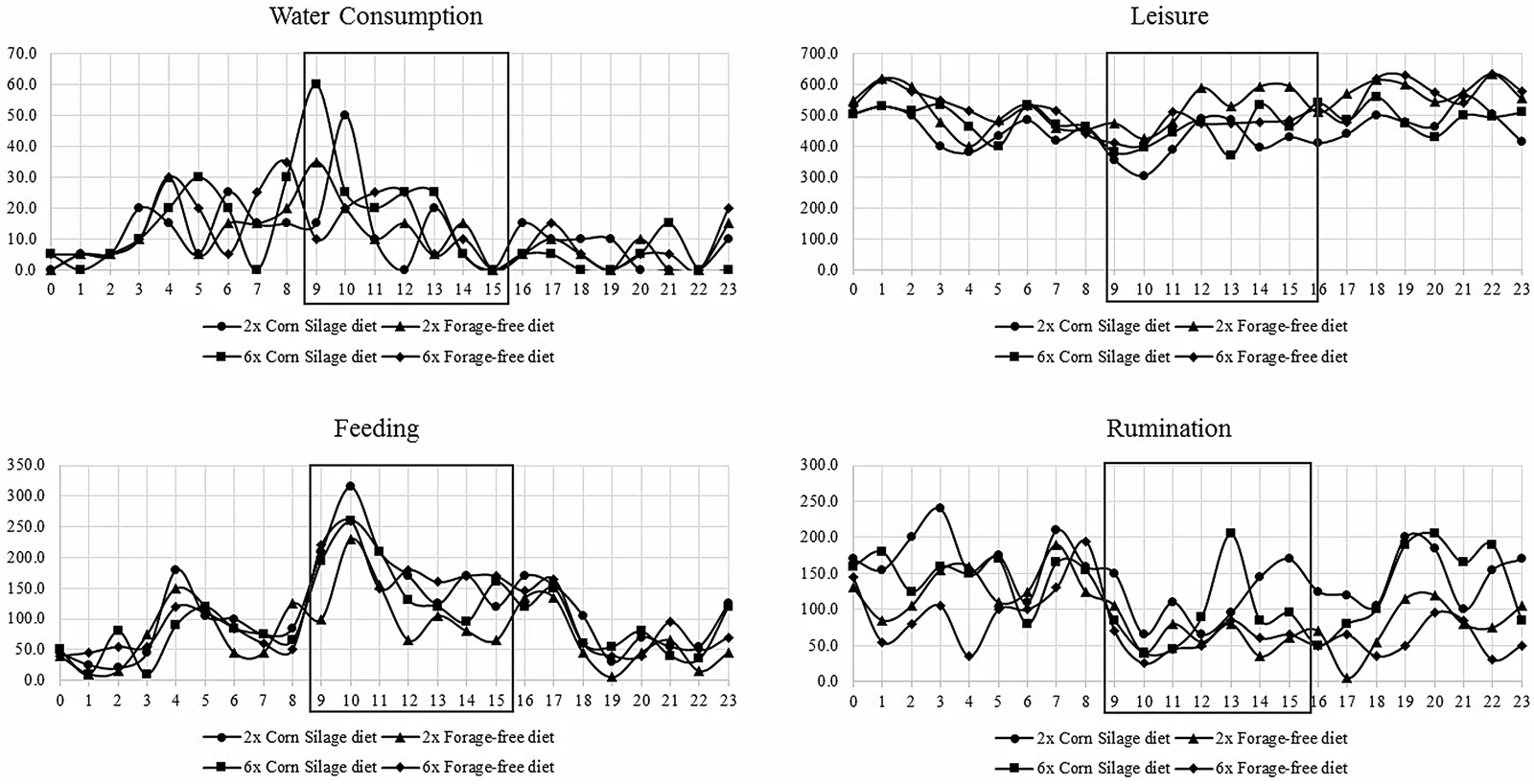
Ingestive behavior of Nellore young bulls finished in confinement according to the dietary fiber source and feeding frequency

Feed intake time was not affected by fiber source or feeding frequency. However, a trend in the interaction between these factors was observed for the variables measured during the diet feeding period (*P* = 0.07 and *P* = 0.06, respectively). The lower averages were seen in animals fed a forage-free diet twice daily during 24-hour evaluation (160 min) and during feed delivery (68 min). Consumption efficiency was significantly influenced by the interaction between the factors (*P* = 0.03), with the highest value in animals fed a diet without roughage, containing cottonseed cake as the fiber source, and fed twice a day during 24-hour evaluation (4.2 kg/h) and during feed delivery (10 kg/h). Rumination time (min and %) was significantly higher in animals fed a diet with roughage (*P* ≤ 0.02), averaging 275 min over 24 h and 61 min during the feeding period (Fig. 1). Rumination efficiency was affected by the fiber source (*P* ≤ 0.02), being greater in animals fed a diet without roughage during 24-hour evaluation (1.35 vs. 0.75 kg/h) and during feed delivery (6.5 vs. 3.70 kg/h) than animal fed diet with roughage (Table 5).

**Table 5:** Ingestive behavior of Nellore young bulls finished in confinement according to the dietary fiber source and feeding frequency

	Treatment				SEM	*P*-value		
	Diet with roughage		Forage-free diet			FS	FF	FS×FF
	Twice times per day (2x)	Six times per day (6x)	Twice times per day (2x)	Six times per day (6x)				
24-hour evaluation								
Rumination (min)	295^a^	255^a^	190^b^	150^b^	28.0	0.001	0.17	0.96
Rumination (%)	20.5^a^	17.7^a^	13.1^b^	10.5^b^	1.91	0.001	0.17	0.96
Feeding (min)	230^a^	201^ab^	160^c^	216^a^	22.4	0.23	0.55	0.07
Feeding (%)	16.0ª	13.9^ab^	11.1^c^	15.0^a^	1.63	0.23	0.55	0.07
Consumption efficiency (kg/h)	2.69^b^	2.83^ab^	4.22^a^	2.27^b^	0.52	0.34	0.08	0.05
Rumination efficiency (kg/h)	0.75	0.79	1.39 ^b^	1.26 ^b^	0.24	0.01	0.83	0.68
Assessment during delivery (8:30 to 15:30)								
Rumination (min)	67.1 ^a^	55.4 ^a^	40.8 ^b^	37.9 ^b^	8.5	0.02	0.40	0.61
Rumination (%)	16.0 ^a^	13.2 ^a^	9.7 ^b^	9.0 ^b^	2.00	0.02	0.40	0.61
Feeding (min)	106ª	92.9^ab^	67.9^c^	104^a^	12.1	0.27	0.36	0.05
Feeding (%)	25.3ª	22.1^ab^	16.2^c^	24.7^a^	2.90	0.27	0.36	0.05
Consumption efficiency (kg/h)	5.60^Bb^	5.97^Bb^	9.96^Aa^	5.25^Ab^	1.12	0.11	0.06	0.03
Rumination efficiency (kg/h)	3.36 ^b^	4.02 ^b^	7.21 ^a^	5.84 ^a^	1.13	0.02	0.76	0.39

FS: fiber source, FF: feeding frequency; FS × FA: fiber source × feeding frequency interaction; SEM = standard error of the mean

^#^ Means followed by a lowercase letter on the same line differ from each other by the F test for feeding frequency and means followed by an uppercase letter on the same line differ from each other by the F test for fiber source

For biochemical parameters, no significant effects of fiber source, feeding frequency, or the interaction between these factors were observed on blood glucose, AST, creatinine, and total protein concentrations (*P* > 0.10) at both the start and end of the experimental period. There was no significant difference in the variables glucose, AST, GGT, creatinine, and total protein (*P* > 0.10) at the beginning of the experiment. By the end of the experimental period, GGT enzyme activity was affected only by feeding frequency (*P* = 0.04), with higher averages in animals fed six times a day (16.5 uL vs. 10.6 uL). A trend toward a negative correlation between GGT activity and ADG was also noted (r = *− 0*.41; *P* = 0.06). Plasma urea concentration was significantly higher at the end of confinement (*P* = 0.03) in animals fed a diet without forage (55 vs. 43.1 mg/dL), regardless of feeding frequency (Table 6).

**Table 6:** Blood parameters of Nellore young bulls finished in confinement according to the dietary fiber source and feeding frequency

	Treatment				SEM	*P*-value		
	Diet with roughage		Forage-free diet			FS	FA	FF×FA
	Twice times per day (2x)	Six times per day (6x)	Twice times per day (2x)	Six times per day (6x)				
Start of confinement								
Glucose (mg/dL)	46.5	53.0	63.7	48.6	9.70	0.50	0.65	0.26
Aspartate aminotransferase (uL)	54.2	49.4	51.4	68.5	12.1	0.63	0.76	0.48
Gamma-glutamyltransferase (uL)	11.4	10.1	8.0	10.0	3.10	0.56	0.92	0.59
Plasma urea concentration (mg/dL)	16.6	15.1	16.1	17.6	1.90	0.58	0.99	0.42
Creatinine (mg/dL)	1.73	1.50	1.70	1.90	0.17	0.67	0.58	0.72
Total protein (g/dL)	4.65	3.80	4.50	4.06	1.00	0.96	0.50	0.82
End of confinement								
Glucose (mg/dL)	86.3	87.5	73.9	89.3	11.9	0.62	0.43	0.50
Aspartate aminotransferase (uL)	87.4	89.3	69.6	88.3	11.0	0.40	0.36	0.45
Gamma-glutamyltransferase (uL)	12.3	15.5	9.0	17.5	2.9	0.83	0.04	0.34
Plasma urea concentration (mg/dL)	42.1^b^	44.1 ^b^	55.8 ^a^	54.3 ^a^	5.7	0.03	0.96	0.74
Creatinine (mg/dL)	2.05	2.05	2.28	2.00	0.18	0.53	0.53	0.53
Total protein (g/dL)	6.43	6.47	6.43	6.18	0.53	0.79	0.84	0.79

FS: fiber source, FF: feeding frequency; FS × FA: fiber source × feeding frequency interaction; SEM = standard error of the mean

^#^Means followed by a lowercase letter on the same line differ from each other by the F test

Similarly, there was no significant effect of supply frequency or interaction with fiber source on economic variables (*P* ≥ 0.63; Table 7). Revenue (U$$/animal) did not differ based on the fiber source (*P* = 0.85), with mean values of U$1,033 for diets with corn silage and U$1,021 for those with cottonseed cake. The diet containing corn silage had higher feed costs (U$188/animal vs. US$112/animal; *P* = 0.003) and, as a result, a lower margin (US$659/animal and US$769/animal; *P* = 0.02).

**Table 7:** Economic performance of Nellore young bulls finished in feedlot according to dietary fiber source and feeding frequency

	Treatment				SEM	*P*-value		
	Diet with roughage		Forage-free diet			FS	FA	FF×FA
	Twice times per day (2x)	Six times per day (6x)	Twice times per day (2x)	Six times per day (6x)				
Revenue (US$/animal)	1,054	1,012	1,028	1,014	59.8	0.85	0.64	0.81
Food cost (US$/animal)	202 ^a^	175 ^a^	114 ^b^	110 ^b^	17.9	0.003	0.10	0.85
Margin (US$/animal)	654 ^b^	665 ^b^	743 ^a^	796 ^a^	42.3	0.02	0.45	0.63

FS: fiber source, FF: feeding frequency; FS × FA: fiber source × feeding frequency interaction; SEM = standard error of the mean

^#^Means followed by a lowercase letter on the same line differ from each other by the F test

## Discussion

The lack of significant differences in performance between fiber sources may be attributed to the similar levels of DMI. According to Mertens (1997), between 60% and 90% of the variation in animal performance is attributed to intake, while only 10% to 40% is related to digestibility. Both groups (diet with roughage and forage-free diet) consumed diets with 37.5% NDF, higher than the average of 21.8% in Brazilian feedlots (Silvestre and Millen 2021). Furthermore, the peNDF was similar between both diets: 75.7% of the cottonseed cake and 76.3% of the corn silage were retained on particles larger than 8 mm, which ensured adequate stimulation of rumination, chewing, salivation, and ruminal buffering (Mertens 1997). Studies by Goulart et al. (2020b). Niwa et al. (2023); Arcanjo et al. (2024b) also demonstrate that cottonseed cake is capable of providing peNDF at levels compatible with maintaining ruminal health, even in diets free of roughage.

Although diets have adequate levels of peNDF, the higher rumination time and rumination efficiency for animals fed roughage can be due to the larger rumen volume occupied by this feed, which increases filling and, in turn, stimulates rumination. On the other hand, animals fed a diet containing cottonseed cake as a fiber source had shorter rumination time. According to Mertens (1997) and Van Soest (1994), this behavior is related to the physical effectiveness of the fiber present in cottonseed cake, which contains a lower proportion of long particles and, despite stimulating rumination, requires less time in rumination. These results are consistent with the findings of Arcanjo et al. (2024b), who also observed greater feed efficiency and reduced rumination time in diets composed of this byproduct.

Regarding feed frequency, the superior performance of animals fed twice daily, with an additional gain of 29.0 kg in TWG and 0.4 kg/day in ADG, may be due to the greater efficiency with which nutrients are utilized when feeding is less frequent, provided the diet contains adequate levels of peNDF. Furthermore, dividing feed intake throughout the day, particularly due to excess stimulation due to the high daily supply causing physical fatigue, in addition, the intermediate supplies were carried out in the hottest hours of the day while the animals fed twice a day did not have this stimulation, thereby negatively affecting performance. It should be noted that the maximum temperatures observed during the confinement period exceeded 35 °C. Silva et al. (2018) observed a similar result, finding no significant effect of feeding frequency (one to four times a day) on the intake or CY of confined Nellore cattle.

Analysis of blood parameters (glucose, AST, GGT, creatinine, and total protein) showed that these remained within the physiological limits showed by Kaneko et al. (2008). This suggests that the metabolic homeostasis of Nellore young bulls was not affected by the different fiber sources or feeding frequency management. These findings corroborate those of Arcanjo et al. (2024a), who also reported metabolic stability in cattle fed cottonseed cake.

The low concentrations of glucose, AST, urea, and total protein observed at the beginning of the experiment can be attributed to the animals’ previous nutritional status. Before the trial, the animals were maintained on dry pasture, which provided limited nutrient availability, particularly in terms of energy and protein. This nutritional deficiency likely resulted in reduced metabolic activity and lower blood metabolite levels. As the experiment progressed and the animals received the experimental diets by 76 days with improved nutritional balance, these parameters increased as dietary effect, independent of the treatment, even though blood samples were collected under fasting conditions at both sampling times.

Higher GGT enzyme levels may be related to productive performance, as a negative correlation was observed between its activity and weight gain. The highest GGT activity was recorded in animals fed six times a day, which also showed a tendency for lower ADG, suggesting greater hepatic stimulation resulting from diet fractionation; however, no clinical changes were observed. Nevertheless, all values remained within the normal range, confirming that replacing roughage with cottonseed cake as a fiber source, combined with automated diet provision, is a viable nutritional strategy from a physiological perspective.

It has been reported that whole cotton plant waste is a favorable source of fiber for small ruminants and can substitute up to 40% of common forages (or 20% of diet DM) without any adverse effects on the growth performance and blood metabolites of feedlot lambs (Kazemi and Tohidi 2023).

Economically, cottonseed cake has emerged as a viable alternative to corn silage. As an agro-industrial by-product, it has lower acquisition costs, is easy to store, can be mixed to produce a complete ration, and can be supplied automatically, all of which directly impact reduced operating costs and increase the economic margin per animal. Studies by Arcanjo et al. (2024b) and Paulino et al. (2013) reinforced this advantage by highlighting that forage-free diets simplify management in feedlots. Bellengeri et al. (2019) also identified silage management as one of the main logistical obstacles in intensive systems. Furthermore, because it is a widely available ingredient in the Central-West region, the use of cottonseed cake contributes to the sustainability of the production system, reducing pressure on areas dedicated to forage production (Goulart et al. 2020a).

Thus, the hypothesis that cottonseed cake is a viable source of non-forage fiber in concentrate-rich diets to feedlots is confirmed, promoting performance equivalent to that of corn silage, with lower feed costs and greater operational viability. On the other hand, the hypothesis that increasing the frequency of feeding the diet improves production performance is rejected, as no significant effects of this variable were observed in the present study.

## Conclusion

Cottonseed cake is a viable alternative as a fiber source in a roughage-free ration for feedlot beef cattle, contributing to the sustainable intensification of the finishing system. This substitution does not compromise the animals’ productive performance or carcass characteristics, demonstrating equivalence with corn silage. A sixfold increase in the frequency of daytime feeding does not provide additional benefits in nutrient intake, productive performance, or economic performance.

## Data Availability

The datasets generated and/or analyzed during the current study are available from the corresponding author in reasonable request.
